# Sustainable Cost Models for mHealth at Scale: Modeling Program Data from m4RH Tanzania

**DOI:** 10.1371/journal.pone.0148011

**Published:** 2016-01-29

**Authors:** Emily R. Mangone, Smisha Agarwal, Kelly L’Engle, Christine Lasway, Trinity Zan, Hajo van Beijma, Jennifer Orkis, Robert Karam

**Affiliations:** 1 FHI 360, Durham, North Carolina, United States of America; 2 Gillings School of Global Public Health, University of North Carolina at Chapel Hill, Chapel Hill, North Carolina, United States of America; 3 School of Nursing and Health Professions, University of San Francisco, San Francisco, California, United States of America; 4 Kenan-Flagler School of Business, University of North Carolina at Chapel Hill, Chapel Hill, North Carolina, United States of America; 5 Text to Change, Amsterdam, The Netherlands; 6 Johns Hopkins Center for Communication Programs, Baltimore, Maryland, United States of America; University Hospital Oldenburg, GERMANY

## Abstract

**Background:**

There is increasing evidence that mobile phone health interventions (“mHealth”) can improve health behaviors and outcomes and are critically important in low-resource, low-access settings. However, the majority of mHealth programs in developing countries fail to reach scale. One reason may be the challenge of developing financially sustainable programs. The goal of this paper is to explore strategies for mHealth program sustainability and develop cost-recovery models for program implementers using 2014 operational program data from Mobile for Reproductive Health (m4RH), a national text-message (SMS) based health communication service in Tanzania.

**Methods:**

We delineated 2014 m4RH program costs and considered three strategies for cost-recovery for the m4RH program: user pay-for-service, SMS cost reduction, and strategic partnerships. These inputs were used to develop four different cost-recovery scenarios. The four scenarios leveraged strategic partnerships to reduce per-SMS program costs and create per-SMS program revenue and varied the structure for user financial contribution. Finally, we conducted break-even and uncertainty analyses to evaluate the costs and revenues of these models at the 2014 user volume (125,320) and at any possible break-even volume.

**Results:**

In three of four scenarios, costs exceeded revenue by $94,596, $34,443, and $84,571 at the 2014 user volume. However, these costs represented large reductions (54%, 83%, and 58%, respectively) from the 2014 program cost of $203,475. Scenario four, in which the lowest per-SMS rate ($0.01 per SMS) was negotiated and users paid for all m4RH SMS sent or received, achieved a $5,660 profit at the 2014 user volume. A Monte Carlo uncertainty analysis demonstrated that break-even points were driven by user volume rather than variations in program costs.

**Conclusions:**

These results reveal that breaking even was only probable when all SMS costs were transferred to users and the lowest per-SMS cost was negotiated with telecom partners. While this strategy was sustainable for the implementer, a central concern is that health information may not reach those who are too poor to pay, limiting the program’s reach and impact. Incorporating strategies presented here may make mHealth programs more appealing to funders and investors but need further consideration to balance sustainability, scale, and impact.

## Introduction

The practice of medicine and public health supported by mobile devices (mHealth) is rapidly being adopted and mainstreamed into all areas of health programming.[1] Due to high penetration of mobile phones in low-resource settings and the availability of short message service (SMS) on all mobile phones, text messages are increasingly used to deliver health information to low-resource populations.[2,3] A growing body of evidence indicates that mobile phones have the potential to improve access, knowledge and healthy behaviors in the context of sexual and reproductive health.[4–11] However, despite the proliferation and promise of mHealth interventions, the majority of mHealth interventions in developing countries fail to continue beyond the pilot stage or reach scale.[12,13] One reason for this may be the difficulty of developing sustainable financial strategies and securing ongoing funding for mHealth programs.[14,15]

A systematic review of mHealth programs attributed the success of many of these programs to the relatively low cost of mobile technology.[10] However, limited data on the cost of programs at scale, mechanisms for financial sustainability, and the cost-effectiveness of these programs have stymied efforts to convince industry and government partners to invest resources in nationally scaled mHealth programs.[10,15] One study that examined the cost-effectiveness of using text messages to improve health workers' adherence to malaria case-management guidelines in Kenya at scale found that in each scenario, costs were relatively low.[16] The intervention was cost-effective at USD 0.03 per correctly managed child.[16] A more limited evaluation of a small scale program in India found that the cost to the National AIDS Control Program (NACP) of sending mobile phone reminders to improve adherence to Anti-Retroviral Treatment (ART) among people living with HIV was also relatively inexpensive.[17] This program cost USD $1.27-$1.77 per patient per year, and the projected total cost of the SMS reminder program accounted for only 0.36% of the total five-year national program budget.[17] While the results of these studies are encouraging, empirical data on the cost of mHealth programs at scale and on strategies for financial sustainability are needed to strengthen the evidence base for the financial feasibility of mHealth interventions at scale and to persuade decision makers to mobilize adequate resources.

Mobile for Reproductive Health (m4RH) is a mobile phone health intervention that has successfully scaled in Tanzania. m4RH is an automated, on-demand SMS system that provides essential information about nine different contraceptive methods to address the limited knowledge, misconceptions, fears and health concerns surrounding contraception. These factors have resulted in high levels (50–81%) of unmet need for modern contraception in East Africa.[18–20] Designed by FHI 360 and with initial funding from the US Agency for International Development (USAID), m4RH was piloted in Tanzania in 2010 and scaled nationally as a free service in 2014. Text to Change (TTC) was the m4RH technology partner in Tanzania. TTC built and operated the technology platform and negotiated with mobile network operators (MNOs) for the short telephone number, or short code, used to access the program. TTC also negotiated with MNOs regarding the price to send and receive SMS. To access m4RH content, users text “m4RH” to short code 15014 to receive a welcome message and content menu, after which users can send a code via SMS (e.g. “11” for contraceptive implants) and the system automatically responds with information about the selected method. For each SMS sent by a user, users typically receive between one and three SMS in response, depending on the number of characters needed to provide the requested information.

This paper presents the complete program costs of national-level implementation of m4RH in Tanzania in 2014. m4RH has a robust user base in Tanzania, and use of the program continues to increase. Program costs are entirely supported by the donor, and there is keen interest to explore other revenue models to sustain the program. Therefore, we use the current program costs to explore three strategies for the financial sustainability of m4RH: revenue generation, SMS cost reduction and key partnership development. The overall aim of this paper is to facilitate a discussion on the sustainability of mHealth interventions at scale.

## Methods

Our analyses have two main objectives: (1) to present a detailed description of the costs of implementing a national SMS-based behavior change communication service like m4RH in Tanzania; and (2) to explore four scenarios for recovering program costs to inform a strategy for sustainability.

### Program Costs

For the first objective, we used 2014 program costs, user volume, and SMS traffic data (the number of SMS sent and received by m4RH users) from m4RH records for Tanzania to present a detailed description of the costs. Program costs are broken down into technology costs, administration costs, and personnel costs. Technology costs include server costs, short code fees, network fees, and technical support. Administration costs include quarterly data reporting, project management and communication, and SMS cost. The number of users (i.e. user volume) were sourced from TTC records. Promotional costs included advertisements for m4RH in Johns Hopkins Center for Communication Programs’ (CCP) implementation of a national media campaign for family planning. Costs for promoting m4RH were estimated as 10% of their overall media campaign costs ($189,000) and were paid for by CCP. Personnel costs were pulled from FHI 360 program budgets. Costs are presented from the perspective of the program implementer and are reported in 2014 US Dollars (USD).

When providing the description of the m4RH program costs, cost components were further categorized as either fixed costs or variable costs. We defined fixed costs as not varying, or varying minimally, with the volume of users and variable costs as costs that were tied to the number of users. Fixed costs included technology, administrative, personnel, and promotional costs. Variable costs included SMS costs. Cost per user was calculated based on all fixed and variable costs for 2014.

### Strategies for Cost Recovery

**Fig 1:**
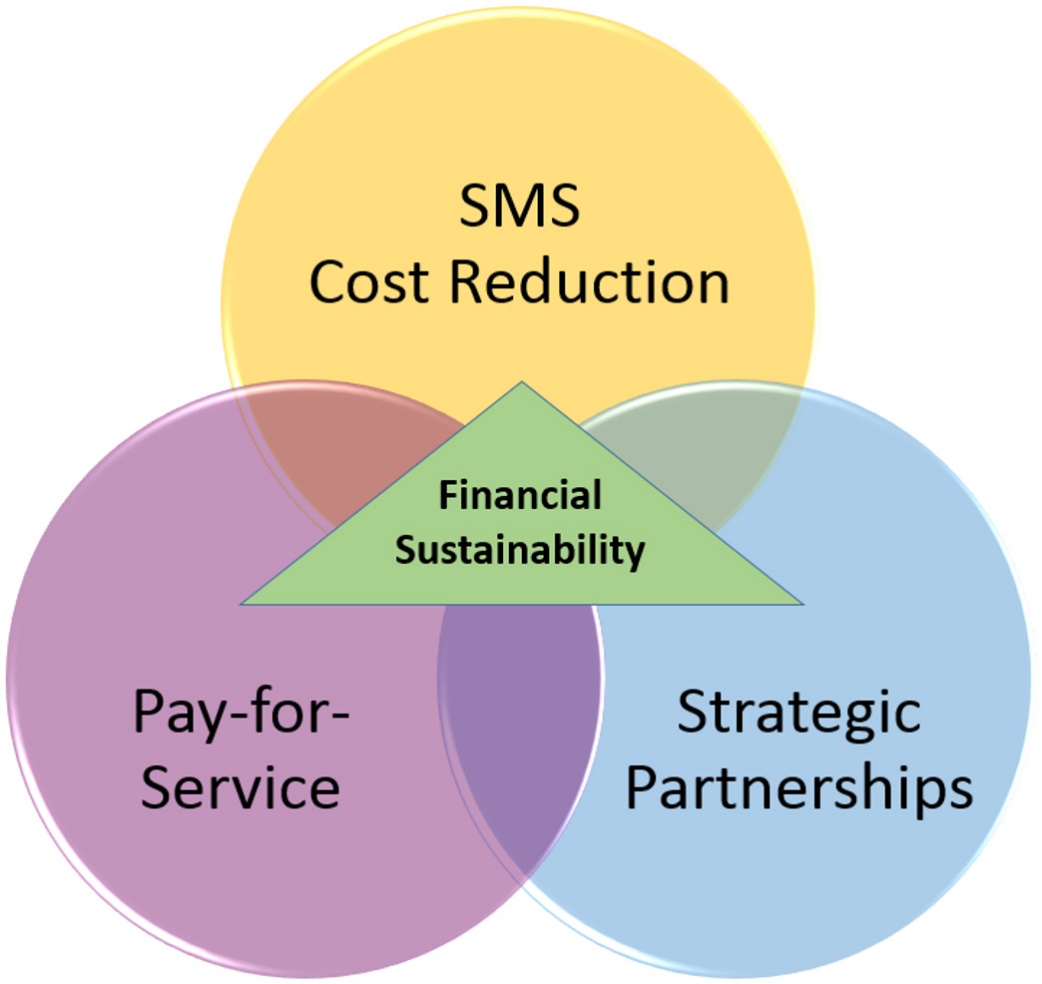
Framework for Financial Sustainability for mHealth Programs.

For the second objective, we explored multiple strategies to support cost recovery. Our initial discussion reflected two broad approaches—cost reduction and revenue generation—and we identified specific options under each approach. Initial revenue generation options included charging individuals user fees, pushing advertisements to users, partnerships with private health care organizations, and offering anonymous user data to interested organizations. Initial cost reduction options included partnering with alternative technology partners, investigating alternative technology platforms, fully transferring ownership of m4RH to the Ministry of Health, and leveraging multi-channel marketing opportunities. We then developed decision trees to guide our reflection about each option. After consulting with a range of stakeholders including mobile network operators, local technology aggregators, local health care groups, and technology-savvy business consultants about the feasibility of these approaches, we eliminated several options and subsequently focused our framework on three key strategies. These are: (1) user pay-for-service, (2) SMS cost reduction, and (3) strategic partnerships. Our vision is that these three strategies for cost recovery are synergistic and that the intersection of these strategies may lead to program financial sustainability, as illustrated in Fig 1.

User pay-for-service means that the user pays for all or a portion of the cost of the service they receive. In the context of an SMS service, users can pay for text messages they send (standard SMS cost-structure in Tanzania), pay for messages that they send and receive, pay subscription fees to receive or access messages from a specific content provider, or pay a tiered or premium cost for more valuable content which would subsidize free content. For the purpose of simplicity, we considered only scenarios in which users either pay for messages that they send, or pay for both the messages that they send and receive. Because users of programs like m4RH are often resource-constrained, we set the SMS cost at the 2014 standard SMS rate in Tanzania, which was USD $0.03 (48TZS) per SMS, and did not consider using SMS costs higher than standard rates.

The SMS cost reduction strategy is based on the assumption that reduced SMS rates can be negotiated with the mobile network operator (MNO). Reduced SMS costs could be negotiated with increased user volume (that consequently yield increased revenue to the MNO), as part of a larger partnership, or as part of a pre-packaged “bundle” of services.[21–23] Another mHealth program in Tanzania called *Wazazi Nipendeni* (Parents Love Me) has successfully established public-private partnerships with MNOs that have resulted in a reduced SMS cost of $0.01 per SMS.[23] This cost structure would allow the health program to realize a small, per-SMS profit margin if users are charged standard rates while the program pays the negotiated bulk SMS price.[22] We used this cost structure for four break-even analyses to determine the profit or loss under each scenario. We estimated two different reduced SMS rates; a reduced rate of $0.02 per SMS to the m4RH program in Scenarios 1 and 2, and $0.01 per SMS to the m4RH program in Scenarios 3 and 4. In all scenarios we maintained a price of $0.03 per SMS for the m4RH user. This resulted in either $0.01 (Scenarios 1 & 2) or $0.02 (Scenarios 3 & 4) per-SMS profit margins for the m4RH program.

The third strategy we explore is leveraging key partnerships. Several configurations of partnerships can be created leading to cost-reductions, as described in the context of Wazazi Nipendeni above. As another example, the MAMA Bangladesh program called Aponjon established partnerships with multiple MNOs to develop revenue sharing terms, waivers for registration charges, and unrestricted choices of networks for family members.[21] In the context of m4RH, in 2012 FHI 360 established a memorandum of understanding (MOU) with CCP to advertise m4RH as part of an existing mass media campaign. Under this partnership, advertisements for m4RH were included as part of CCP’s ongoing communication campaign. This cost-sharing arrangement meant that m4RH only paid for minimal promotional costs, though the full promotional costs are still included in the budget. In our modeling activities and analysis, we used this specific strategic partnership, and the resulting reduced promotional costs. Implicit partnership agreements were also assumed as part of the SMS cost reduction strategy (e.g. with MNOs to reduce the per SMS cost) based on our review of partnerships established by other programs. Additional partnership strategies are presented in the discussion.

### Break-Even Analysis

We used different configurations of the three proposed strategies to generate and explore the financial sustainability of four scenarios (Table 1). In all scenarios, strategic partnerships were leveraged to reduce costs. In Scenarios 1 and 2, m4RH negotiated a reduced bulk rate of $0.02 per SMS. This led to $0.01 profit margin per SMS when users paid for messages. In Scenarios 3 and 4, m4RH negotiated a reduced bulk rate of $0.01 per SMS leading to a $0.02 profit margin per SMS when users paid for messages. In Scenarios 1 and 3, users paid only for the messages that they sent to m4RH (standard SMS cost structure in Tanzania; 8 SMS based on 2014 program data). Thus the program realized profit margins only on these messages. In Scenarios 2 and 4, users paid both for the messages that they sent to m4RH and for the messages that they received back (32 SMS based on 2014 program data). Thus the program realized profit margins for all messages sent and received.

**Table 1:** Four Scenarios for Financial Sustainability**.

	Pay for Service		Cost Reduction	Sustainability Margin
Scenario	(Cost to user per SMS sent by user)	(Cost to user per SMS received by user)	(Cost to m4RH per SMS sent or received by user)	Per SMS Profit Margin
1	$0.03	$0.00	$0.02	$0.01*
2	$0.03	$0.03	$0.02	$0.01
3	$0.03	$0.00	$0.01	$0.02*
4	$0.03	$0.03	$0.01	$0.02

*Per SMS profit margin only realized on messages sent by users.

**Partnership is implicit in every scenario

For each of the scenarios shown in Table 1, we calculated two outcomes: 1) the net profit/cost to the program at 125,320 users (the total number of unique m4RH users in 2014), and 2) the total number of users required to recuperate program costs (i.e. break-even user volume). The first outcome holds user volume and promotional costs constant based on program data in 2014. The analysis for the second outcome assumes that user volume will increase and decrease proportionately with promotional activities and uses the Excel Solver function to identify the number of users at which program revenue will equal program cost. For this analyses, promotional costs were treated as variable because increased promotion correlates with increased user volume and SMS traffic. We divided the total promotional cost ($19,400) by the total number of users in 2014 (125,320) to obtain a promotional cost of $0.15 per user.

### Uncertainty Analysis

Because our models are based on only one year of data we expect that the number of users, the average number of SMS that users send and receive from m4RH (SMS traffic), and the amount spent on promotional costs may fluctuate from year to year. We therefore conducted a probabilistic uncertainty analysis around these three key program variables to determine whether our break-even results were sensitive to variations in these inputs and what the range of expected profit or cost might be. We used Crystal Ball to run a 10,000 trial Monte Carlo simulation. Promotional cost per user and per-user SMS traffic were assigned normal distributions with means of $0.15 and 32 SMS, respectively, and standard deviations of $0.02 and 2 SMS, respectively. Program data from 2014 indicated that the average number of texts sent and received per user was 32 SMS. Because this was the number of informational messages accessed by users when the program was completely free, we used 32 SMS as the average number of SMS that sufficiently answers users’ questions about contraception, family planning, and reproductive health in order to calculate the average cost of m4RH content to users. User volume was assigned a lognormal distribution because of its positive skew, with a mean of 125,320 (the user volume from 2014). These values and distributions for the uncertainty analysis were selected based on limited one-year data presented in the results section and expectations of future program costs and user volumes.

## Results

### Program Costs

Program costs are presented in Table 2. Implementation of m4RH in Tanzania in 2014 cost $203,475 and engaged 125,320 unique users. This represented a program cost of $1.62 per user. Overall annual SMS traffic was 4,057,190 SMS with 25% of all SMS being sent from users to the m4RH server (1:3 ratio). On average, users sent eight text messages to the m4RH system and received twenty-four messages back (an average of 32 SMS per user). Over half of total program costs (63%) were attributed to SMS costs because m4RH paid for the cost per SMS ($.032) for all m4RH messages sent and received by the user. Administration and technology costs were the next largest budget expenses, $23,040 and $21,000, respectively. Promotional costs accounted for approximately ten percent of the budget ($19,400). Personnel costs were the smallest budget item at $11,707.

**Table 2:** 2014 m4RH Program Costs for Tanzania.

Cost Type	Cost Description	2014 USD
**Annual Fixed Costs ($75,147)**		
Technology costs	Server costs ($7,800)	$21,000
	Short code fees ($3,000)	
	Network fees ($4,200)	
	Technical support ($6,000)	
Administration costs	Quarterly data report ($600)	$23,040
	Project management & communication ($22,440)	
Personnel costs	20% salary for local manager ($6,000)	$11,707
	5% salary for US-based advisor ($5,705)	
Promotional Costs	$18,900 (10% of mass media campaign of $189,000)*	$19,400
	Medium intensity paper promotional costs: $500	
**Annual Variable Costs ($128,328)**		
SMS costs	*[SMS cost] x [# of SMS sent/user] x [# of users]*	$128,328
	SMS cost: 0.032 per SMS	
	Average SMS sent to/from m4RH user: 32	
	Number of unique users: 125,320	
**Total 2014 m4RH Program Costs:**		**$203,475**

*Cost-shared by CCP

### Break-Even Analysis

Table 3 presents the results from the four break-even analyses. In Scenario 1 there was a $94,596 budget shortfall (cost) to m4RH when we set the user volume to 125,320 users. We also examined whether there was a certain user volume at which program costs would break even with program revenue. However, because per user promotional costs were higher than the per user revenue, there was no break-even point. As a result, the budget shortfall only increased with additional users. In Scenario 2, there was a $34,443 budget shortfall for the program when we set the user volume to 125,320 users. However, program costs broke even with program revenue at 327,924 users and additional users led to a profit under the same structure. In Scenario 3, there was an $84,571 budget shortfall at 125,320 users and again there was no point at which the program revenue would break even with program cost because of the relatively high promotional costs. In Scenario 4, it would only take 113,769 users for program costs to break even with program revenue, so with the 2014 total of 125,320, there was a $5,660 profit. Any additional users would lead to a greater profit for the program.

**Table 3:** Results from Four Break-Even Scenarios.

Scenario	Cost to user per SMS	Cost to m4RH per SMS	M4RH program profit or cost (-) at 125,320 users	Users needed for costs to break-even with revenue	Average total cost to users
1	$0.03*	$0.02	-$94,596	Not possible	$0.16
2	$0.03	$0.02	-$34,443	327,924	$0.64
3	$0.03*	$0.01	-$84,571	Not possible	$0.24
4	$0.03	$0.01	$5,660	113,769	$0.96

*Users pay only for SMS they send to m4RH

Table 3 also presents the average total cost to users for m4RH content. In the four scenarios above, the average total cost to users was $0.16 (8 SMS * $0.02) in Scenario 1, $0.64 (32 SMS * $0.02) in Scenario 2, $0.24 (8 SMS * $0.03) in Scenario 3, and $0.96 (32 SMS * $0.03) in Scenario 4.

### Uncertainty Analysis

The uncertainty analyses showed that the results of the breakeven analyses were fairly sensitive to uncertainty around user volume, per user SMS traffic, and SMS promotional cost per user, with results being most sensitive to user volume (Table 4). The average profit/loss outcomes for Scenarios 1–4 were very similar to the outcomes in the original analyses: program losses of -$94,683, -$34,459, -$84,639, and a profit of $5,457, respectively. However the range for the 5^th^ and 95^th^ percentiles for trials around those means were fairly broad, indicating that uncertainty around expected user volume makes it difficult to get a precise estimate of program whether the program will realize a profit or loss. In Scenarios 1 and 3 all expected outcomes were budget shortfalls (costs). In Scenario 2, the budget shortfall was smaller, and in 0.02% of trials we would expect m4RH to realize a profit. In Scenario 4, the average outcome was a profit and we would expect that in repeated trials, 62% of the time revenue would equal or exceed cost for this scenario.

**Table 4:** Results of Monte Carlo Probabilistic Uncertainty Analysis.

Scenario	Mean m4RH Program Profit or Cost (-)	5^th^ Percentile (Lower Bound)	95^th^ Percentile (Upper Bound)	Difference between 5^th^ & 95^th^ Percentiles	% of trials that break even or realize a profit
1	-$94,683	-$109,710	-$83,608	$26,102	0%
2	-$34,459	-$42,168	-$24,141	$18,027	<1%
3	-$84,639	-$96,231	-$76,168	$20,063	0%
4	$5,457	-$12,655	$29,798	$42,453	62%

## Discussion

Strategies for sustainable and cost-efficient scale up of mHealth interventions are urgently needed to ensure that the benefits of mobile technology in public health are realized at scale. A necessary first step toward establishing a financially sustainable business model is having an accurate understanding of the costs of operating these types of mHealth interventions. To date, few mHealth interventions have published or otherwise made available information regarding their program costs. This limits the ability of current and future program implementers or funders to understand cost structures and to explore pathways to financial sustainability. In this paper, we have presented the costs associated with national-level implementation of an mHealth program for improving family planning knowledge and use in Tanzania. With this data, we then applied three interrelated strategies for cost recovery to explore potentially sustainable financial models.

With an overall annual program cost of $203,475 for a nationally scaled program—or $1.62 per person directly reached with family planning information— m4RH represents a reasonable cost to a donor like USAID, which had a 2014 budget of nearly $621 million for reproductive health and family planning.[24] However, donor funding does not represent a truly sustainable financial model as it can fluctuate significantly. One could argue that a country government could support a program such as m4RH but it may be fairly expensive given that the total Tanzanian per capita health expenditure, for example, is only $49 (2013).[25]

Many mHealth interventions struggle with ensuring consistent and sufficient financial resources over the long-term. Some interventions, like HNI’s 3-2-1 service, were able to partner with Mobile Network Operators (MNOs) for a short period to obtain free SMS or voice services for users, thereby reducing the program costs considerably.[26] Other interventions, such as MAMA Bangladesh, add fee-for-service schemes to strategic partnerships to further reduce costs.[27] Sustainability options must match the specific characteristics of the mHealth intervention. In the case of m4RH, where the majority of our costs are represented by SMS fees, we opted to explore other means of shifting SMS charges to users (e.g. pay for service) and reducing program costs through partnerships (e.g. reduced SMS rates).

From the four scenarios and the sensitivity analysis, we found that breaking even (and realizing a profit) was only probable when all SMS costs were transferred to the user and the lowest per-SMS cost ($0.01) was negotiated with partners (Scenario 4). In Scenario 4, the total average cost to the user to receive m4RH services was $0.96 per user, which may be high in the context of the Tanzanian per capita GDP of $695 (2013).[28] While this model for sustainability was the least expensive from the perspective of the implementer, a central concern is that the family planning health information would not reach those who were too poor to pay for the service, limiting the program’s reach and impact.

Although Scenarios 1–3 did not achieve a zero cost outcome for program implementers at the 2014 number of users, they did represent large reductions in annual program cost: 54%, 83%, and 58%, respectively. Additionally, the total average cost to the user of accessing m4RH represented lower barriers to entry than Scenario 4 at $0.16, $0.64, and $0.24, respectively. Incorporating the strategies presented here and reducing the cost of the program implementation may make mHealth programs like m4RH more appealing to governments and other donors. The government could also act as a key partner in helping to negotiate reduced costs, promoting the service as part of its existing promotional campaigns, or integrating the program into an existing service.

While we limited our analysis to three strategies, it may be possible to recover additional costs by considering alternative strategies such as data-mining, pushing targeted marketing (advertising) to m4RH users, bundling m4RH with other mHealth services to achieve economies of scale, or partnering with interested for-profit parties such as insurance companies that may be willing to pay for all or part of the cost of m4RH in order to provide a value-added service to their customers. The practical and ethical implications of some of these strategies, including offering anonymous data to paying organizations, data-mining, and targeted advertising, should be further explored before they are implemented as revenue generating strategies in mHealth programs. Other costing strategies could include tiered payments, subscription fees for all you can use services, and offering the first 6 messages (basic content) free to users after which users would pay for services.[26] Although we initially explored such strategies, for this paper we focused on pay for service and SMS cost reduction approaches that appear to be most feasible based on other implementers’ successes with these strategies and on our stakeholder consultations.

As with all models, there are limitations to our analysis. The most critical of these limitations is the assumption that users will pay for health communication messages via SMS. While paying to send text messages is standard practice in Tanzania, charging users to receive content may limit program use: in a willingness-to-pay survey of 1,332 m4RH users, less than half (47%) of users were willing to pay for the service (data not shown). Especially in a resource-constrained setting, transferring costs to users may deter program use for the poorest and most vulnerable segments of the population. However, some studies have indicated that paying a fee may increase user investment in the program and the perception that the program is providing a valuable service.[29] While the mHealth field is still experimenting with this approach, more programs are seeing success by balancing reach with reduced program costs and we can expect to see more mHealth services experimenting in this way.[26,27]

A second limitation is that we only used data for one year of the program so we were unable to capture program variability and trends over a longer period of time. Understanding fluctuations in user volume and program use would certainly be important for developing a strategy for financial sustainability. However, we limited our study to 2014 because that is the year that the program scaled nationally and therefore combining program statistics with earlier years may have skewed program figures.

A final limitation in this field is the dearth of cost and cost-effectiveness data on these types of programs which would allow direct side-by-side comparisons of intervention impact and could bolster support for mHealth interventions among funders and potential investors.[14,15] Without reliable data on the effectiveness of an intervention, decision makers are constrained in their ability to make evidence-based choices regarding what programs to fund.

## Conclusion

The intent of this analysis was to model program costs and a basic set of strategies for cost recovery in order to initiate a conversation about how to sustainably finance mobile phone interventions at scale in the context of family planning and reproductive health. This analysis raises several questions with respect to the use of SMS as a communication format for large scale health information dissemination and behavior change. While we present strategies for program costs to break even with program revenue, strategies to recover costs alone may not be sufficient for sustainability. Cost recovery does not allow a margin to cover unexpected costs, account for increases in fixed costs, or to make any additional improvements in the system. In the short-term, donor funds are likely to remain an essential component of mHealth services targeting the poor and underserved in LMICs but strategies such as the ones presented in this paper can help to reduce the total amount of required donor funding. For long-term sustainability, the global mHealth community—including donors—must begin to discuss incorporating successful and socially desirable business models that can lead to profit generation. Profits generated by mHealth programs can be reinvested in the programs to improve, innovate, and grow these services, with the ultimate goal of increasing their health impact.

## Supporting Information

S1 Supporting Information Excel File of Breakeven Analysis and Monte Carlo Simulation. (XLSX)
